# Three-needle of regulating the mind combined with umbilical needle intervention for cervical vertigo with deficiency of qi and blood—A study protocol for a randomized controlled trial

**DOI:** 10.3389/fneur.2025.1476596

**Published:** 2025-06-18

**Authors:** Zhuanzhuan Hou, Jun Tian, Yuewei Jin, Huida Yu, Weigang Wu, Miaomiao Wei, Shibing Xu

**Affiliations:** ^1^Department of Acupuncture and Moxibustion, The Third People's Hospital of Xiaoshan District, Hangzhou, China; ^2^Department of Orthopedics, The First People's Hospital of Xiaoshan District, Xiaoshan Affiliated Hospital of Wenzhou Medical University, Hangzhou, China

**Keywords:** cervical vertigo, deficiency of qi and blood, three-needle of regulating the mind, umbilical needle, randomized controlled trial

## Abstract

**Objective:**

With a higher incidence rate among the younger population, cervical vertigo (CV) can impose a significant burden on patients with CV. It is believed that a deficiency of qi and blood is the primary pathogenesis in Traditional Chinese medicine (TCM). Acupuncture is a vital strategy for CV, but the efficacy of current acupuncture therapies remains unsatisfactory. Thus, this randomized controlled study will assess the safety and effectiveness of three-needle of regulating the mind combined with umbilical needle (TNRM-UN) therapy for CV with qi and blood deficiency.

**Methods:**

This was a single-center, assessor-, and analyst-blinded, prospective randomized controlled trial that involved treatment for 6 weeks and follow-up for 24 weeks. Ninety-nine patients with CV deficiency of qi and blood are randomly allocated to three groups in a 1:1:1 ratio. The patients in the observation group will receive three-needle of regulating the mind combined with umbilical needle (TNRM-UN) therapy. In contrast, the patients in the control-1 group will receive three-needle of regulating the mind combined with traditional acupuncture (TNRM-TA), and the patients in the control-2 group will receive only umbilical needle (UN) therapy. Primary outcomes include Cervical Vertigo Symptom and Functional Assessment Scale (CVS-FAS) score and qi and Blood Deficiency Symptoms Assessment Scale (QBD-SAS) score. The secondary outcomes are hemodynamic changes in the bilateral vertebral arteries, including diameter value (DV), peak systolic velocity (PSV), end-diastolic velocity (EDV), pulsatility index (PI), and resistance index (RI). All outcome measurements will be evaluated before and after 6 weeks of treatment. Primary outcomes will be evaluated at 12 and 24 weeks of follow-up. Adverse events (AEs) were evaluated during treatment and follow-up periods.

**Results:**

This study aimed to determine whether TNRM-UN is more effective than current acupuncture therapies by providing compelling evidence and deciding whether TNRM-UN can be used as a promising therapy for CV with a deficiency of qi and blood.

**Clinical trial registration:**

https://www.chictr.org.cn/, identifier: ChiCTR2400080759.

## Introduction

Cervical vertigo (CV) is a broad term encompassing dizziness caused by various cervical pathologies. In contrast, cervical spondylosis-induced vertigo refers to dizziness resulting from degenerative changes in the cervical spine, such as osteophyte formation or intervertebral disc herniation. This study focuses on the latter, as defined by the inclusion criteria. CV is associated with various symptoms such as disequilibrium, light-headedness, tinnitus, nausea, migraine, and neck pain or stiffness (1, 2). It has been reported that the estimated prevalence of CV is between 3.8% and 17.6%. Approximately 50% of patients with cervical spondylosis have vertigo symptoms (3), while 65%−66% of vertigo in elderly patients is attributed to cervical spine dysfunction (4). TCM considers that middle-aged and elderly people suffer from CV mostly due to insufficient kidney essence and deficient qi blood biochemistry. Therefore, this study is specifically concerned with CV related to qi and blood deficiency. In recent years, with the widespread use of electronic devices, the onset of CV issues has occurred at a younger age, and the risk of developing stroke and syncope is higher (5). Therefore, major academic circles are paying more attention to this disease, and it is particularly important to explore its pathogenesis, diagnostic ideas, and more effective treatment methods.

So far, modern medicine has believed that the pathological mechanism of CV primarily involves the following theories: (1) Abnormal proprioceptor theory (6). (2) sympathetic nerve theory (7), (3) vertebral artery theory (8), (4) migraine-related theory (9), and (5) neurohumoral factor theory (10). In addition, with the progression of the disease, patients will gradually develop psychiatric factors such as anxiety and depression, which may reduce the patients' self-regulatory function and make the clinical efficacy unsatisfactory (11). Multiple mechanisms, with a dominant mechanism, usually act together in CV. Currently, to address these pathological mechanisms, the main treatment options for CV include drug and surgical therapy in modern medicine. Drug treatment primarily aims to enhance microcirculation, eliminate aseptic inflammatory reactions, and alleviate muscle tension (12). Surgical treatment is mainly to relieve sympathetic nerve stimulation, eliminate mechanical compression of the vertebral artery, and rebuild the stability of the cervical spondylosis segment (13). However, drug treatment has high recurrence and certain toxic side effects, while surgical treatment has the disadvantages of high risk, high cost, and immature surgical techniques, which affect the efficacy (14).

The TCM categorizes CV as “cervical paralysis” and “vertigo.” TCM believes that the pathogenesis of CV is mainly due to a deficiency of qi, blood, and the brain medulla. These mechanisms are similar to the theory proposed by modern medicine that CV is caused by an insufficient blood supply to the vertebral arteries. In addition, patients with CV are often accompanied by emotional problems such as restlessness of the heart and mind and depression of liver qi, which makes it difficult to cure the disease. In recent years, TCM has achieved satisfactory results in treating CV. TCM therapies mainly involve Chinese herbal medicine, manual repositioning, acupuncture, massage, cervical traction, and needle-knife therapy (15–20). However, the experimental designs of most clinical trials were not rigorous enough, and it was insufficient for evidence-based medical thinking. These TCM methods (15–20) only have only shown their effectiveness for this disease, and there were shortcomings such as longer treatment course, slower apparent effect, and higher recurrence rate in clinical practice. The author has done a systematic review and meta-analysis (21) that has supported the efficacy of acupuncture for CV. The author has followed Professor Howard Chen's cervical vertigo treatment team in clinical practice and experimental research for decades has found that treating CV with qi and blood deficiency is closely related to the regulation of the governor vessel and conception vessel. In recent years, the author has successively established “Dredging governor vessel therapy (22)” and “Dredging governor-regulating conception vessel acupuncture method (23)” to treat CV with deficiency of qi and blood. However, there were still the following shortcomings: (1) Acupuncture therapies brought more pain to patients because of puncturing more acupoints, (2) these therapies did not consider emotional factors, and (3) there were no relevant statistics to long-term efficacy.

To avoid these shortcomings, the efficacy, safety, and cost-effectiveness of TNRM-UN will be compared to those of TNRM-TA or UN through a rigorously designed randomized controlled trial, which will provide a clinical basis for using TNRM-UN for CV with a deficiency of qi and blood.

## Methods and design

**Figure 1:**
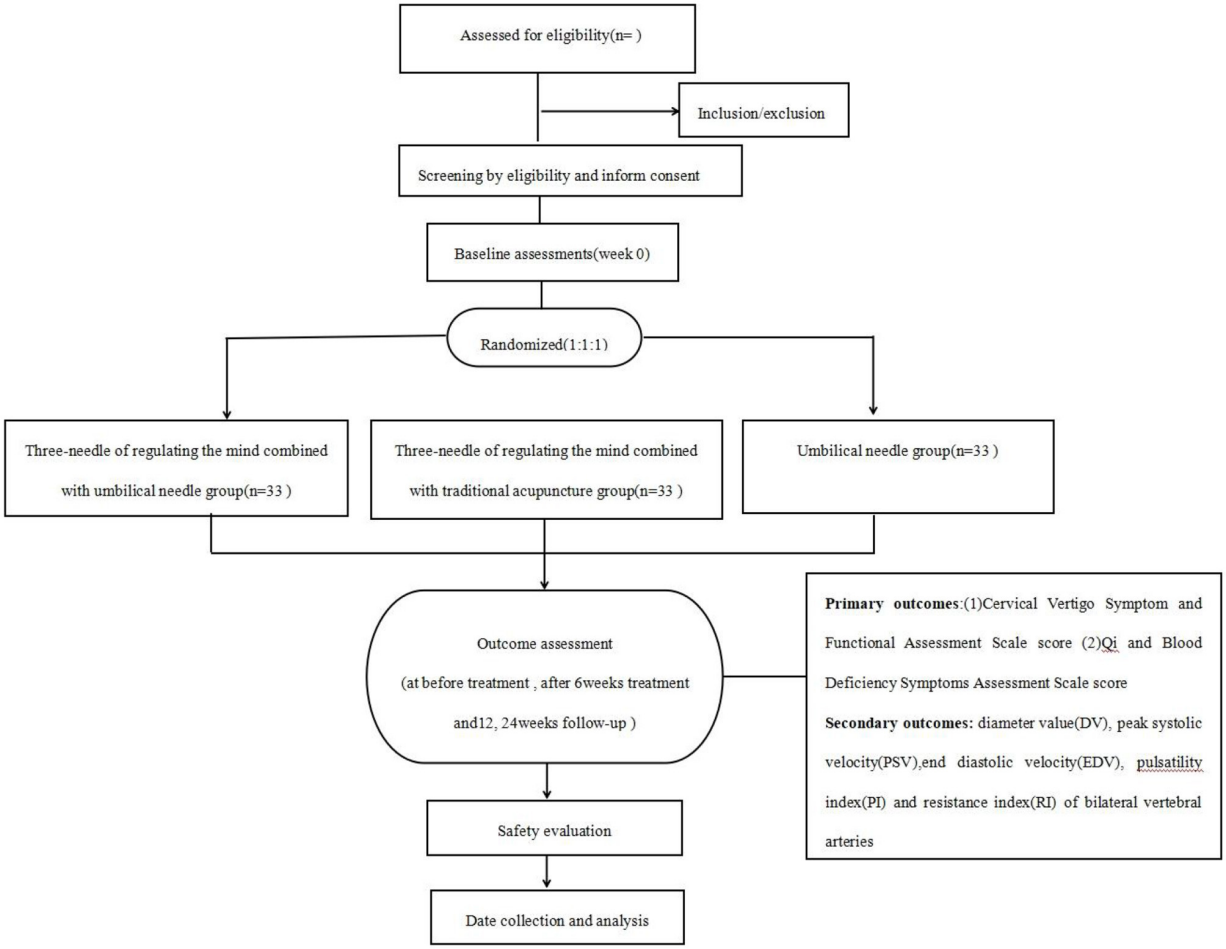
Flowchart of this study.

This is a single-center, assessor- and analyst-blinded randomized controlled trial including three parallel groups, which are TNRM-UN, TNRM-TA, and UN groups. The trial will follow the guidelines of the Standards for Reporting Interventions in Clinical Trials of Acupuncture (STRICTA) (24) and the Standard Protocol Items: Recommendations for Interventional Trials (SPIRIT) (25). This study will include 6 weeks of treatment and 24 weeks of follow-up. The study period was July 2023 to December 2025. Figure 1 presents a flowchart of the trial, and Table 1 shows all schedules.

**Table 1:** Schedules of this study.

	**Baseline**	**Treatment (weeks)**	**Follow-up (weeks)**	
**Time point**	**Week 0**	**Week 6**	**Week 12**	**Week 24**
**Enrolment**				
Eligibility screening	O			
Informed consent	O			
Allocation	O			
**Intervention**				
TNRM-UN		O		
TNRM-TA		O		
UN		O		
**Outcome assessments**				
(1) CVS-FAS	O	O	O	O
(2) QBD-SAS	O	O	O	O
(3) VA-DV	O	O		
(4) VA-PSV	O	O		
(5) VA-EDV	O	O		
(6) VA-PI	O	O		
(7) VA-RI	O	O		
Safety evaluation		O	O	O

O, required; TNRM-UN, three-needle of regulating the mind combined with umbilical needle; TNRM-TA, three-needle of regulating the mind combined with traditional acupuncture; UN, umbilical needle; CVS-FAS, Cervical Vertigo Symptom and Functional Assessment Scale; QBD-SAS, qi and Blood Deficiency Symptoms Assessment Scale; VA-DV, vertebral artery diameter value; VA-PSV, vertebral artery peak systolic velocity; VA-EDV, vertebral artery end diastolic velocity; VA-PI, vertebral artery pulsatility index; VA-RI, vertebral artery resistance index.

### Participant recruitment

Participants will be mainly recruited from the Third People's Hospital of Xiaoshan District, Hangzhou. Outpatients or inpatients from the department of acupuncture and neurology will be recruited. Epidemiological data from our hospital indicated that approximately 60%−70% of CV patients present with deficiency of qi and blood syndrome, aligning with regional demographic trends (26). We have found that 85% of screened patients met the deficiency of qi and blood criteria through screening logs, which ensured sufficient representation. So, recruitment gives priority to CVs with a deficiency of qi and blood. Some news media platforms, including exhibition shelves, the hospital's official account, and relevant newspaper columns, will be used to supplement participant enrollment. These platforms will refer to brief introductions of this study and the researchers' contact information. The researchers will thoroughly document this trial course for every participant and request them to sign an informed consent form.

### Diagnosis criteria

#### Western medical diagnostic criteria

This refers to the diagnostic criteria formulated by the Third National Symposium on Cervical Spine Disease (2008) (27).

(1) Patients had a history of sudden collapse episodes with cervical vertigo.

(2) The neck rotation test was positive.

(3) Patients tend to have cranial symptoms, including blurred vision, tinnitus, and hearing disorders.

(4) Radiographs showing segmental instability or osteophytes of the hook vertebral joint.

(5) Magnetic resonance imaging (MRI) or vertebral artery ultrasonography revealed local stenosis or tortuosity of the second vertebral artery (V–II).

#### Chinese medicine diagnostic criteria

It refers to “TCM diagnostic efficacy criteria” formulated by the Department of Medical Affairs of the State Administration of Chinese Medicine (28).

(1) In mild cases, dizziness or vision rotation can be stopped by closing the eyes, but in severe cases, the patient may feel like riding in a car, boat, or even falling down.

(2) CV may be accompanied by nausea and vomiting, tinnitus, deafness, sweating, and pallor.

(3) CV has a chronic onset that progressively worsens, an acute onset, or recurrent attacks.

#### Classification criteria of qi and blood deficiency

It refers to the syndrome differentiation basis of qi and the blood deficiency type formulated by the internal Medicine of TCM (29).

(1) Vertigo occurred during exertion and was exacerbated during exercise.

(2) Pale complexion and lack of gloss.

(3) Heat palpitates, sleepless, tired, and lazy.

(4) Faint tongue and weak pulses.

### Inclusion criteria

(1) The diagnostic criteria for CV in Chinese and Western medicine and the classification standard for qi and blood deficiency.

(2) Age 20 ≤ age ≤ 75 years, regardless of sex.

(3) Participants could adhere to the therapy plan and did not concurrently participate in other clinical trials.

(4) The participants voluntarily signed informed consent forms.

### Exclusion criteria

(1) Participants diagnosed with other TCM types of CV.

(2) Participants diagnosed with vertigo of heart, brain, and ear origin.

(3) Participants with combined cardiac, cerebral, renal, and other serious primary illnesses.

(4) Participants with abnormal blood flow parameters in segments I and III of the vertebral.

### Discontinuation or elimination criteria

(1) Participants were mistakenly included in the trial.

(2) Participants experienced serious adverse events (AEs) and did not consistently participate in the study.

(3) Participants suffered serious complications during the course of the trial, which required emergency care.

(4) Participants with personal factors were asked to stop the trial.

(5) Participants with poor compliance did not adhere well to the study process.

(6) The participants received treatment outside the intervention.

### Randomization and allocation concealment

Participants will be randomly categorized into TNRM-UN, TNRM-TA, or UN groups; the details of the allocation are as follows:

Randomization sequence generation (30): A statistician independent of the research team will generate the randomization sequence using Statistical Package for the Social Sciences (SPSS) version 25.0 (IBM Corp, USA) with a block randomization method. Sequences will be stratified by baseline CVS-FAS score and age to ensure balance between groups. The randomization list is password-protected and stored on a secure server accessed only by an independent statistician.

Allocation concealment protocol (31): Group assignments are sealed in sequentially numbered, opaque envelopes. Each envelope contains a folded card with the group assignment (TNRM-UN, TNRM-TA, or UN). The coordinator, uninvolved in recruitment or outcome assessment, opens envelopes in sequential order only after confirming participant eligibility. Envelopes are stored in a locked cabinet, and an external auditor will access logs weekly.

Preventive Measure of Bias: Participants and outcome assessors are blinded to group assignments. Acupuncturists could not be blinded due to procedural differences, but are instructed not to discuss treatment details. The statistician analyzing outcomes will receive anonymized datasets labeled “Group A/B/C,” and group identities are revealed only after final analysis.

### Blinding method

The participants will not be blinded due to the completely different therapies used in the three groups. Meanwhile, owing to the unique nature of acupuncture, it is unrealistic to turn a blind eye to acupuncture operators. However, manipulation, evaluation, and data analysis will be performed by independent Investigators. The evaluators and statisticians are blinded to the grouping information.

### Intervention

Each group will be divided into 33 participants who received TNRM-UN, TNRM-TA, and UN treatments separately for 6 weeks. The frequency of the intervention is 3 times per week, and the needles are left in place for 30 min.

### Three-needle of regulating the mind combined with the umbilical needle group

#### Regulating the mind therapy

##### Acupoint selection

Referring to previous studies (21, 32, 33), we chose Shenting (DU24), Baihui (DU20), and Naohu (DU-17) to regulate the mind, the locations of which are listed in Table 2. The acupoint positioning standard is referred to as the acupoint positioning standard (34).

**Table 2:** Locations of acupuncture acupoints.

**Acupoints**	**Location**
Shenting (DU 24)	On the head, 0.5 cun directly above the midpoint of the anterior hairline.
Baihui (DU 20)	On the head, 5.0 cun directly above the midpoint of the anterior hairline, at the midpoint of the line connecting the apexes of both ears.
Naohu (DU-17)	On the head, 2.5 cun directly above the midpoint of the posterior hairline, in the depression on the upper border of the external occipital protuberance.
Fengchi (GB20)	In on the nape, below the occipital, on the level of fengfu (DU20), in the depression between the upper ends of the sternocleidomastoid and trapezius muscles.
Jiaji acupoints (EX-B2)	On each side of the back, below the spinous processes from the 1st thoracic vertebra to the fifth lumbar vertebra,0.5 cun lateral to the posterior midline, Select Jiaji acupoints (EX-B2)corresponding to the sixth cervical vertebra.
Pishu (BL20)	On the back, below the spinous process of the 11th thoracic vertebra.1.5 cun lateral to the posterior midline.

##### Procedures

Patients are asked to take the sitting position, and the skin of selected points is routinely sterilized. The used needles are disposable sterile needles with a length of 40 mm and a diameter of 0.25 mm and are produced in Suzhou Hwato Medical Instruments Co., Ltd., China. When acupuncture is applied to Shenting (DU24), the needle is penetrated horizontally for 25–30 mm into Baihui (DU20) so that the needle sensation is spread to the parietal and temporal regions. When acupuncture is applied to Baihui (DU20), the needle is inserted horizontally for 25 mm along the governor vessel so that the needle sensation is spread to the posterior top and temporal regions. When acupuncture is applied to Naohu (DU-17), the needle is inserted horizontally for 8–12 mm. Acupuncturists use the finger-cutting needle method and perform flat reinforcing and reducing maneuvers after producing sensations such as soreness, numbness, or swelling.

#### Umbilical needle therapy

##### Orientation selection

**Figure 2:**
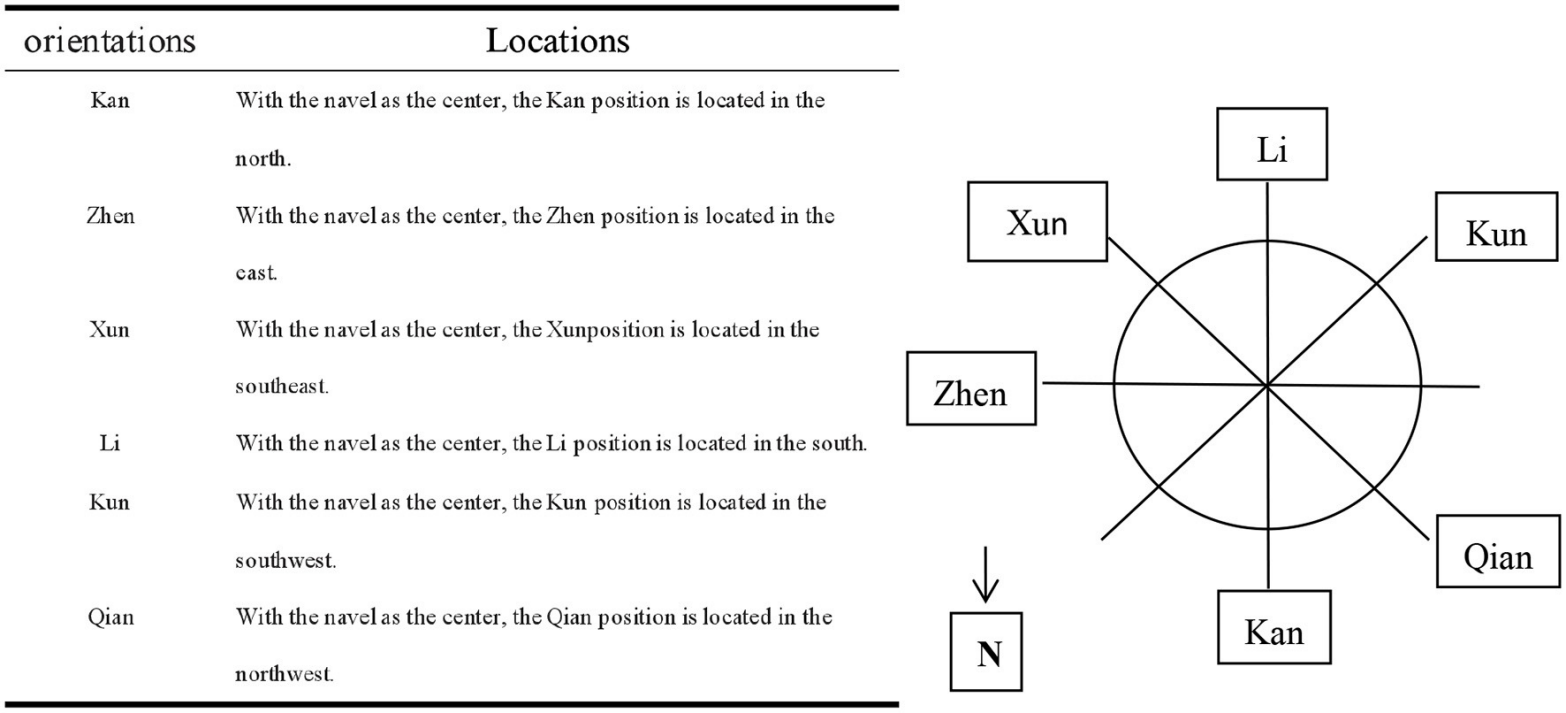
Locating table and schematic of selected umbilical needle orientations.

The positions of Kan, Zhen, Xun, Li, Kun, and Qian are selected according to the hologram of inner eight trigrams, outer eight trigrams, and “Luoshu” in the theory system of umbilical needling (35). Combined with our previous pilot study, acupuncture in these six positions is often effective. Locations of these positions and reference diagram are also summarized in Figure 2.

##### Procedures

**Figure 3:**
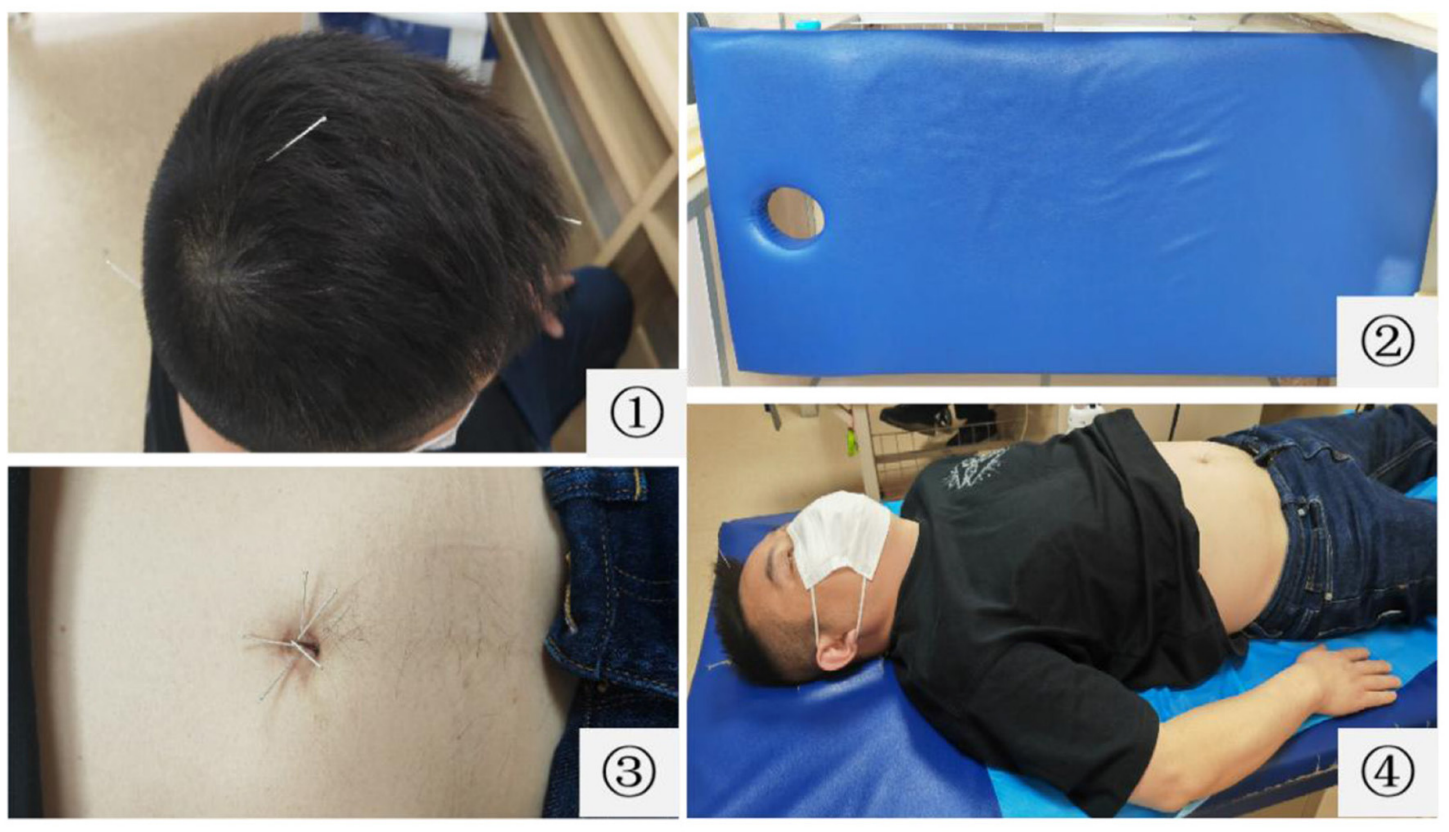
Schematic diagram of three-needle therapy for regulating the mind and umbilical needle therapy. ① Three-needle for regulating the mind; ② acupuncture massage bed with holes at the head of the bed; ③ umbilical needle therapy; ④ three-needle for regulating the mind and umbilical needle therapy.

After regulating the mind therapy, the patients are asked to slowly lie supine on the acupuncture massage bed with holes at the head of the bed, and the area of Naohu (DU-17) point is suspended to ensure the safety of acupuncture. The skin around the navel is routinely disinfected. With the umbilicus core as the center, the disposable sterile needles with a length of 25 mm and a diameter of 0.25 mm are horizontally inserted into the corresponding umbilicus wall for approximately 10 mm at Kan, Zhen, Xun, Li, Kun, and Qian positions in turn. The acupuncturists need to look for sensitive points with pressure and pain near the Kun, Li, Xun, and Qian positions, and when the tip of the needle is aimed at a sensitive point, the doctors use the twirling method to insert the skin, commonly known as a “shotgun.” After needling, the needle handles are adjusted such that they are connected to each other. The operation diagram of TNRM-UN therapy is summarized in Figure 3.

### Three-needle of regulating the mind combined with traditional acupuncture group

#### Regulating the mind therapy

The method is the same as that for the TNRM-UN group, and the patients were asked to be in a prone position because of the need for traditional acupuncture treatment.

#### Traditional acupuncture therapy

##### Acupoint selection

The selected acupoints refer to *Acupuncture and Moxibustion Therapeutics*, (36) which is the recommended textbook for the National Higher Colleges of Traditional Chinese Medicine in the New Century. Primary acupoints involve bilateral Fengchi (GB20) acupoints, bilateral Jiaji (EX-B2) acupoints corresponding to the sixth cervical vertebra, and bilateral Pishu (BL20) acupoints. Locations of these acupoints are also summarized in Table 2.

##### Procedures

Patients are asked to select the prone position. After disinfecting the acupoint skin, the disposable sterile needles with a length of 40 mm and a diameter of 0.25 mm are inserted into the acupoint skin, causing patients to experience a sensation of soreness, numbness, or swelling. The needles are inserted about 15 mm toward the tip of the nose at bilateral Fengchi points. The Jiaji acupoints corresponding to the sixth cervical vertebra are inserted straightly for 12–20 mm. The acupuncture angle of Pishu points is 45° from the body surface, and the depth of the needle is about 20 mm.

##### Umbilical needle group

The method is the same as that used for the TNRM-UN group.

### Outcome measures

All outcome measurements will be assessed before treatment and after 6 weeks of treatment, and primary outcomes will also be assessed at 12, 24 weeks follow-up.

#### Primary outcome measures

(1) Cervical Vertigo Symptoms and Functional Assessment Scale (CVS-FAS) score (37): vertigo degree (8 points), vertigo frequency (4 points), vertigo duration (4 points), neck and shoulder pain degree (4 points), headache degree (2 points), daily life and work ability (4 points), and psychological and social adaptability (4 points).

(2) Qi and blood Deficiency Symptom Assessment Scale score (QBD-SAS) (38): tiredness degree (6 points), palpitations and shortness (6 points), complexion (3 points), tongue substance (3 points), and pulse pattern (3 points).

Lower scores on the above indicators indicate less severe symptoms. Prior to the analysis, the internal consistency of CVS-FAS and QBD-SAS will be evaluated using Cronbach's alpha (α > 0.7 required). Inter-rater reliability is confirmed via Cohen's κ (κ > 0.6) for dual evaluators.

#### Secondary outcome measures

Ultrasound Hemodynamic Assessment Protocol: Bilateral vertebral arteries Hemodynamic parameters including diameter value (DV), peak systolic velocity (PSV), end-diastolic velocity (EDV), pulsatility index (PI), and resistance index (RI) are measured using a Mindray Resona19S ultrasound system (Mindray, Shenzhen, China) with a linear array probe (L14-3WS, frequency: 5–12 MHz). The protocol adheres to international guidelines for vertebral artery assessment. Details of the measurements are standardized as follows:

(1) Patient positioning: Supine position with neck neutral alignment.

(2) Probe orientation: Longitudinal plane, parallel to the vertebral artery's anatomical course.

(3) Gain settings: Adjusted to minimize noise while maintaining vessel wall clarity (default: 60 dB).

(4) Sampling point: Mid-V2 segment (between C4–C6 transverse foramina), with a Doppler angle ≤ 60° and sample gate width 1.5 mm.

(5) Measurement protocol: Three consecutive measurements per side (total 6 per patient). Values from three cardiac cycles are averaged for each parameter (DV, PSV, EDV, PI, and RI). The average values of DV, PSV, and EDV are obtained by taking the arithmetic mean of all valid measurement values of the same parameter. The PI and RI values are calculated by the formulas PI = PSV – EDV/Mean velocity and RI = PSV – EDV/PSV, respectively; they are taken as the arithmetic mean of all valid measurements.

Ultrasound diagnostic criteria for CV (39): VD < 2.80 mm is defined as stenosis; Vmax < 35.00 cm/s, and Vmin < 15.00 cm/s are considered as decreased; RI > 0.70 and PI > 1.30 are considered as increased.

### Sample size estimation

PASS V.15.0 software (NCSS, Kaysville, UT, USA) is used for the sample size calculation, and eligible participants are separated into three groups in a 1:1:1 ratio. Owing to the lack of relevant randomized controlled trials (RCTs) and the exploratory nature of this clinical trial, we have to refer to a formula for estimating the sample size of multiple groups of randomized controlled trials: N = [(*Z*_α/2_ + *Z*_β_)σ/δ] (2) (${\text{Q}}_{1}^{-1}$ + ${\text{Q}}_{2}^{-1}$). Based on pre-experiment research data, after 20 patients received TNRM-UN treatment, the average reduction in CVS-FAS was 7.3 points, with a standard deviation of σ = 8.2; the standard deviation (σ = 8.2) in the formula is directly adopted from the pilot study to reflect population variability. Aligned with clinical consensus for vestibular disorders [Jacobson and Newman have supported using a 30%−50% reduction as clinically meaningful in vestibular outcomes in 1990 (40)], we conservatively set δ (δ = 5 points) as 60% of this observed effect. With α = 0.05, 1 – β = 0.9, and the sample number between groups Q1 = Q2 = 0.5, each group required 30 participants. Assuming a 10% dropout rate, 33 participants need to be included in each group; therefore, in total, we included 99 participants. To ensure robustness, we performed sensitivity analyses evaluating: When effect size (δ) variability was 4–6 points and standard deviation (σ) variability was 7.0 – 9.0, the results confirmed that the planned sample size (N = 99) maintained ≥80% statistical power across all plausible scenarios, supporting its adequacy even if assumptions deviated moderately from initial estimates.

### Statistical analysis plan

Statistical software package SPSS version 22.0 (SPSS, Inc., Chicago, IL, USA) is used for statistical analysis by a blinded statistician. The following content is a detailed introduction to statistical analysis methods:

#### (1) Data distribution assessment

(a) Normality: Evaluated using the Shapiro–Wilk test (*p* ≥ 0.05 required for normality).

(b) Homogeneity of variance: Assessed via Levene's test (*p* ≥ 0.05 required for equal variances).

#### (2) Between-group comparisons

(a) Parametric approach: For normally distributed data with homogeneous variances, one-way analysis of variance (ANOVA) is performed. If the omnibus test is significant (*p* < 0.05), *post hoc* pairwise comparisons are conducted using Tukey's HSD to control the family-wise error rate (FWER).

(b) Non-parametric approach: For non-normal or heteroscedastic data, the Kruskal–Wallis test is applied, followed by Dunn's test with Bonferroni-adjusted *p*-values (α = 0.017).

#### (3) Within-group comparisons

Paired *t*-tests (normal data) or Wilcoxon signed-rank tests (non-normal data) are used to evaluate pre-post changes.

#### (4) Sensitivity analysis

(a) Bonferroni correction: Directly applied to pairwise *t*-tests (adjusted α = 0.017).

(b) Multiple imputation: Missing values are imputed via chained equations (MICE algorithm) with 20 imputed datasets, incorporating baseline covariates and post-dropout outcomes (e.g., adverse events), which will validate results under missing-at-random assumptions. We add psychological status (baseline Hospital Anxiety and Depression Scale [HADS] scores), pain threshold (quantitative sensory testing [QST] for pressure pain tolerance at the trapezius muscle), and sociodemographic factors (age, sex, and education level) as the baseline covariates mentioned here to adjust for reliability of the subjective outcome measures.

### Safety evaluation

Acupuncture-associated adverse events (AEs) include intolerable pain, bleeding, local hematoma, and other uncomfortable feelings after acupuncture (e.g., pale face, cold, moist limbs, cold sweat, dizziness, nausea, rapid heartbeat, weak pulse, and even fainting). All AEs, which will be considered as unrelated, possibly related, or related to treatment, will be recorded and assessed by investigators on an adverse event. Investigators should record in detail the occurrence date, degree [mild, moderate, or severe (41)], duration of symptoms, and emergency measures of AEs.

Timely rescue measures will be taken for serious adverse events. Simultaneously, researchers will immediately report to the Ethics Committee, which will decide whether to withdraw the participants from the study.

To comprehensively assess delayed-onset AEs and acupuncture safety, we will take the following measures: (1) Extended follow-up duration: Participants will receive monthly telephone follow-ups for 6 months post-trial completion (up to week 48) to capture delayed-onset AEs. (2) Patient-reported outcome diaries: Participants will document daily symptoms (e.g., pain and numbness) in a standardized diary for 12 weeks post-treatment. (3) Electronic Health Record (EHR) Linkage: With participants' consent, we will access electronic health records for a 1-year post-trial to identify their hospitalizations or diagnoses potentially linked to acupuncture.

Standardized Criteria for AE Causality Assessment will adopt the following measures: (1) WHO-UMC Causality Criteria (42): (a) Certain: AE occurs post-treatment, aligns with known acupuncture effects, and resolves upon discontinuation. (b) Probable/likely: Temporal association with no confounding factors. (c) Possible: Plausible link, but alternative explanations exist. (d) Unlikely: No plausible connection to treatment. (2) Naranjo algorithm (43): A quantitative tool to score causality likelihood (scores: ≥5: “probable”; ≤ 1: “doubtful”). (3) Independent adjudication: A blinded Safety Review Board (SRB) comprising a neurologist, TCM expert, and pharmacovigilance specialist will resolve disputed cases.

### Ethical approval and study registration

The Ethics Committee of the Third People's Hospital of Xiaoshan District, Hangzhou, granted ethical approval (Xiaoshan Third Hospital 2022 Research No.: 004) for carrying out this study. Prior to enrollment, each participant will be informed of detailed information about the study, welfare, and possible risks. All participants will be allowed to withdraw from this study at any time according to their consent. Written informed consent must be obtained from all participants, whose privacy will be strictly protected. The study protocol has been registered in the Clinical Trials Registry under the identification code ChiCTR2400080759.

### Data collection and management

Independent researchers collect raw data for all participants using printed case report forms (CRFs) and enter them into an electronic case report form (eCRF). Physical, printed version of the document CRFs will be kept in a locked filing cabinet in the primary researcher's work office. In contrast, electronic data will be locked in a password-protected computer. Only authorized data managers and statisticians can access these files, and both the physical, printed version of the documents and electronic research documents will be preserved for at least 5 years after publication.

### Quality control

To ensure acupuncturist training and procedural consistency, acupuncturists with at least 5 years of clinical experience need to undergo a 4-week centralized training program prior to the trial. The curriculum included: (1) Theoretical modules: (a) TCM principles of qi and blood deficiency and cervical vertigo pathogenesis and (b) anatomical landmarks for acupoint localization. (2) Practical skill standardization: (a) Needling techniques (depth, angle, and retention time), for TNRM-UN, TNRM-TA, and UN therapy, guided by the Standards for Reporting Interventions in Clinical Trials of Acupuncture (STRICTA) and (b) simulation training using three-dimensional (3D) anatomical models to ensure precision in acupoint targeting. (3) Assessment criteria: (a) Skill examination: Blind evaluation of needle insertion accuracy (±2 mm tolerance) and manipulation (e.g., needle insertion technique, “De Qi” elicitation). (b) Theoretical test: A minimum score of approximately 80% on a 100-item exam covering TCM theory and intervention protocols.

Regarding ultrasound operator training and consistency, we will adhere to under quality control: (1) Operator training: Two sonographers with ≥5 years of neurovascular experience performed all measurements. They underwent centralized training to standardize probe placement, angle correction, and data acquisition. (2) Intraoperator consistency: A subset of 20 patients undergo repeated measurements by the same operator (48-h interval). Intraclass correlation coefficients (ICC) for DV, PSV, EDV, PI, and RI are 0.92–0.96, indicating excellent repeatability. (3) Interoperator consistency: For 20 randomly selected patients, both operators independently perform blinded measurements. Interoperator ICCs ranged 0.88–0.94. ICC reference standards (44) are shown in Table 3.

**Table 3:** Interoperator and intraoperator consistencies of hemodynamic measurements.

**Parameter**	**Intra-operator ICC (95% CI)**	**Inter-operator ICC (95% CI)**
Diameter (DV)	0.94 (0.89–0.97)	0.91 (0.85–0.95)
PSV	0.96 (0.92–0.98)	0.94 (0.89–0.97)
EDV	0.92 (0.86–0.96)	0.88 (0.81–0.93)
PI	0.93 (0.87–0.96)	0.89 (0.82–0.94)
RI	0.95 (0.90–0.97)	0.90 (0.83–0.95)

ICC, intraclass correlation coefficient; CI, confidence interval; PSV, peak systolic velocity; EDV, end-diastolic velocity; PI, pulsatility Index; RI, Resistance Index.

The implementation of the trial will be checked periodically during the study period. All study materials, including the original data and the occurrence of AEs or complications, will be collected in CRFs. We will employ a data monitoring committee to verify the authenticity and consistency of the raw data with the recorded data. The principal investigator will organize regular meetings to discuss problems that arise during the study and put forward optimal solutions.

Finally, to enhance recruitment and adherence of the participants, all treatment expenses were free of charge to participants, and during the follow-up period, we will improve patient compliance through appropriate financial compensation and health education.

## Discussion

### Research status of Acupuncture for CV

Cervical vertigo (CV) is a common disease related to cervical spine dysfunction. Acupuncture, a traditional Chinese medicine therapy, has attracted much attention in recent years due to its safety, effectiveness, and multitarget regulation characteristics. The following three aspects are a summary of the research status at home and abroad from three aspects: Clinical efficacy, mechanism of action, and research characteristics. (1) Clinical efficacy: A recent domestic randomized controlled trial (RCT) by Li et al. (45) used Fengchi (GB20) as the main acupoint for acupuncture treatment of CV. After treatment, the patients' ESCV scores were better than those before treatment (*p* < 0.05). The results indicated that acupuncture treatment for cervical vertigo can improve patients' quality of life. A 1-year follow-up study (46) in the United States (46) showed that the recurrence rate of CV in the acupuncture group (28%) was significantly lower than that in the drug group (55%), with no severe adverse reactions. (2) Mechanism of action: Domestic studies (47, 48) have revealed that acupuncture can relieve the spasm of the vertebral artery, stimulate the sympathetic and parasympathetic nerves, and improve microcirculation to improve blood supply to the brain. Domestic studies believe that regulating vertebral artery blood circulation system is the primary mechanism of acupuncture in the treatment of CV. The mechanism of foreign research focuses on regulating proprioception, neural, and humoral factors. Luo et al. (49) have also revealed that acupuncture can improve proprioceptive disorders by improving the connection between the proprioceptors and the brain, and establishing the correct pattern of sensation and movement in the organism. Jiang et al. (50) concluded that the adequate stimulation of acupoints by acupuncture can reduce the content of neuropeptide Y (NPY) in serum, effectively improve the blood circulation of the vertebral artery, and then improve the symptoms of vertigo. (3) Research focus: Domestic research emphasizes the overall syndrome differentiation and acupoint compatibility, and pays attention to the curative effect of integrated traditional Chinese and Western medicine. Foreign studies focus on standardized protocols and neurophysiological mechanisms and emphasize placebo control.

In the clinical treatment of CV, drug and surgical treatment have difficulty obtaining satisfactory results, and some inevitable side effects often limit their efficacy. Research by Xu et al. (51) showed that the total effective rate of acupuncture in treating cervical vertigo was higher than that of Western medicine. Moreover, acupuncture had more advantages than Western medicine in improving the symptoms of cervical vertigo, the Functional Evaluation Scale (ES-CV) score, and the blood supply of the vertebral–basilar artery. Zhu et al. (52) found that the incidence of adverse reactions in patients with cervical vertigo treated with acupuncture was 0.0%, while that in the conventional Western medicine treatment group was 2.5%. This indicates that acupuncture in treating cervical vertigo has a lower risk of adverse reactions. In this context, acupuncture may be a potentially satisfying therapy for CV, but the current evidence is still unsatisfactory due to significant methodological flaws and potential publication bias; moreover, among the numerous acupuncture treatments for CV, which is the most effective treatment, the present study aimed to find a simple, low-cost, safe, and more effective acupuncture treatment method for CV through high-quality research methods.

### Strengths of the study

In TCM, the activities of the mind and spirit depend on transformation of qi and moistening of the blood, if the deficient qi and blood cannot transform mind and carry spirit, it will inevitably lead to the loss of use of the mind and the loss of nourishment of the brain marrow, thus developing vertigo. Therefore, in this study, qi and blood deficiency type is taken as the primary research syndrome of CV. This study suggests, for the first time, that the patient's mind should first be stabilized through the three-needle of regulating the mind. The reasons for choosing Shenting (DU24), Baihui (DU20), and Naohu (DU-17) to regulate patients' minds are as follows: (1) These three acupoints are the essential acupoints of the forebrain, top of the brain, and hindbrain, respectively. Brain acupoints have a shallow muscular layer richly distributed with vessels and nerves. Appropriate acupuncture stimulation at the brain acupoints can converge the lax mind, regulate brain discharge, and release neurotransmitters to improve the function of the brain to achieve the purpose of regulating the mind. (2) The three acupoints belong to the governor vessel that enters the collateral brain on the rooftop. The running course of the governor vessel in the back overlaps with the spinal cord, while, brain is the sea of marrow. A branch of the governor vessel runs through the heart to the throat, so the governor vessel is closely related to the heart. The heart is the birthplace of the mind; therefore, the regulatory effect of the governor vessel on the mind is much greater than that of other meridians. (3) The efficacy of the three acupoints themselves also plays a key role. Shenting (DU24) point can affect people's minds by regulating the governor vessel. Baihui (DU20) is where all Yang meridians meet on the head, which is one of the essential points for regulating brain function. Naohu (DU-17) point is the gateway for the meridian qi of the governor vessel in and out of the brain, which brightens the eyes, relieves spasticity, and calms the mind.

Umbilical needle therapy is a characteristic acupuncture method gradually developed by Professor Qi Yong, through many clinical practices combined with Yi Jing theory, holographic theory, time medicine theory, and embryonic development theory, which also refers to TCM theory, including five elements theory, meridian theory, and organ picture theory (53). Each acupoint in the human body carries information about its own body, and the umbilicus contains the highest information element of the human body due to the particularity of the umbilicus in embryonic development and human anatomy. This study is the first to apply umbilical needle therapy to treat CV. This study selected Kan, Zhen, Xun, Li, Kun, and Qian positions to treat CV for the following reasons: (1) The umbilicus, the location of Shenque (CV8) point, belongs to conception vessel. The conception and governor vessels exit from the uterus, with one source but two different sources, ascending and converging on the head and face, closely related to the brain and spirit. Shenque point (umbilicus) itself regulates the circulation of qi and blood, supplementing essence blood, consolidating the kidney, and cultivating essence (54, 55). (2) According to the holographic law of “luo shu” in the umbilical needle theoretical system, Xun, Li, and Kun positions correspond to the right shoulder, neck, and left shoulder, respectively. TCM believes that the onset of CV is closely linked to the liver, spleen, and kidneys. According to the holographic theory of the inner eight trigrams, the positions of Zhen, Kun, and Kan correspond to the liver, spleen, and kidneys, respectively. According to the holographic theory of outer eight trigrams, the positions of Qian, Kan, and Li correspond to the head, ear, and eye, respectively. So, these positions can not only treat the main symptoms of CV but also treat accompanying symptoms of CV, such as headache, dizziness, blurred vision, tinnitus, etc. Moreover, Kan and Li positions constitute “water-fire combination” to improve spinal instability in these combinations of orientations. Zhen and Xun positions constitute a “thunder-wind struggle” to relieve anxiety and depression caused by dizziness. Kun and Qian positions constitute “earth-day tai” to take the intersection of yin and yang. Zhen, Li, and Kun's positions constitute “three-needle for strengthening the spleen” to improve qi and blood deficiency syndrome.

The innovations in the intervention approach of this study are explained as follows: Regarding the three-needle method of regulating the mind, its theoretical basis is the meridian system theory of TCM, the stimulated meridian qi is acquired meridian qi, the acupuncture site is in the brain, and the acupuncture points are located in the governor vessel. Regarding umbilical needle therapy, its theoretical basis is Yi Jing theory, the stimulated meridian qi is innate, the acupuncture site is in the umbilical region, and the acupuncture point is located in the conception vessel. Umbilical needle therapy breaks through the 1,000 of years confinement of “Shen que forbidding needles” and paves the way for the precedent of “one point curing hundreds of diseases.” This study is the first attempt to combine the two methods in treating CV. This method combines the theory of TCM and Yi Jing, the combination of the human brain and umbilical cord, the combination of the governor vessel and conception vessel, and the combination of the innate and acquired meridians. This method has the advantages of few acupuncture points, simple operation, and less pain. While it appears to be simple, it contains a great deal of theoretical basis and information, and fully embodies the holistic view of TCM and the principle of “the way is simple.”

The innovations in the control setting of this study are explained as follows: (1) The three-needle regulating the mind combined with the traditional acupuncture group was compared to the three-needle regulating the mind combined with the umbilical needle group. Both groups received initial treatment with three needles to regulate the mind for mental relaxation, and there was only a difference in the single factor of “traditional acupuncture” or “umbilical needle.” This study aimed to confirm the superiority of the umbilical needle compared with traditional acupuncture as a control. (2) The three-needle regulating the mind combined with the umbilical needle group was compared with the umbilical needle-only group. Both groups received treatment with an umbilical needle, and there was only a difference in the single factor of whether a three-needle regulating the mind was used. We propose to confirm the importance of using a three-needle to regulate the mind in CV.

### Clinical implications of hemodynamic changes

The hemodynamic parameters measured via color Doppler ultrasound–vertebral artery diameter (DV), peak systolic velocity (PSV), end-diastolic velocity (EDV), pulsatility index (PI), and resistance index (RI) are critical biomarkers for assessing vertebrobasilar insufficiency, a key contributor to cervical vertigo (CV). In clinical practice, increased arterial diameter (DV), peak systolic velocity (PSV), and end-diastolic velocity (EDV) reflect enhanced blood volume and velocity, which are associated with improved cerebral perfusion. Prior studies (39) have shown that PSV < 35 cm/s or EDV < 15 cm/s correlates with symptomatic dizziness, and VD < 2.80 mm is defined as vertebral artery stenosis. Normalization of these values post-intervention predicts reduced CV recurrence. Elevated RI (>0.70) and PI (>1.30) indicate heightened vascular resistance, often linked to arterial stiffness or stenosis. A reduction in these indices post-treatment suggests improved vascular compliance and downstream perfusion, which may alleviate symptoms such as nausea and lightheadedness (56). The clinical prognostic value of hemodynamics is as follows: Sustained hemodynamic improvements (e.g., stable PSV and reduced RI) are associated with long-term symptom remission and lower risk of cerebrovascular complications (57). For instance, patients with persistent PI > 1.30 have a 2.5-fold higher likelihood of recurrent vertigo (58).

In this trial, improvements in these parameters following TNRM-UN therapy may indicate restored vertebral artery flow dynamics, which aligns with symptom alleviation (e.g., reduced CVS-FAS scores). However, individual variability in hemodynamic-symptom correlation warrants cautious interpretation.

## Limitations

(1) The inherent inability to blind participants and acupuncturists in acupuncture trials introduces two key biases: (a) Performance Bias: Acupuncturists may unconsciously intensify treatment for the experimental group (TNRM-UN), inflating observed effects. (b) Reporting Bias: Participants aware of receiving active therapy may overreport symptom improvement, particularly for subjective outcomes. To this end, to mitigate bias partially, we will employ the following strategies:

(a) Standardized participant pre-treatment education: All participants will receive identical oral/written explanations about potential benefits of all interventions (TNRM-UN, TNRM-TA, and UN) to equalize expectations.

(b) Third-party outcome monitoring: Independent assessors will review video recordings of 30% randomly selected acupuncture sessions to ensure protocol adherence.

(c) We will incorporate an electronic health record (EHR) to track long-term outcomes, which can reduce subjective reporting reliance.

(2) Due to the lack of relevant RCTs, our sample size has to refer to the formula for the sample size of multiple groups of randomized controlled trials. The pilot trial's single-arm design (*n* = 20) may not fully capture variability in a three-group RCT. We conservatively set δ as 60% of this observed effect (δ = 5 points), while clinically justified, it lacks direct empirical validation for CVS-FAS, which requires empirical validation in future trials. Nevertheless, our study will provide compelling evidence for sample size estimation for future RCTs.

(3) Deficiency of qi and blood is the predominant TCM syndrome in CV (60%−70% in our hospital), and our study protocol may not apply to patients with other TCM syndromes. But we acknowledge the potential impact of subgroup proportion on generalizability. Future multisyndromic trials are needed to validate these results across broader populations.

(4) About ultrasonic measurements, while our measurement protocols align with the European Society of Neurosonology Guidelines for Extracranial Doppler Ultrasonography, subtle variations in sampling gate positioning or patient motion may affect PI/RI reproducibility. Future studies could employ automated angle-tracking software to minimize variability further.

(5) While this trial includes a 24-week follow-up period to assess mid-term outcomes, we recognize that this duration may not fully capture the long-term sustainability of therapeutic effects or potential delayed adverse events. To address this gap, we plan to conduct an extended follow-up study (up to 48 weeks) for participants who complete the current trial. This extension will focus on: (a) long-term symptom trajectories: reassessing CVS-FAS and QBD-SAS scores at 36 and 48 weeks to determine durability of symptom relief, (b) delayed adverse effects: monitoring for late-onset complications associated with repeated acupuncture, and (c) patient compliance and behavior: Investigating whether patients adopt supplemental therapies post-trial and their impact on outcomes.

(6) We did not select sham acupuncture as the control arm for two reasons: (a) Acupuncture is very popular in hospitals that recruit participants. Many patients have extensive experience with the sensation of acupuncture, which makes the blinding of participants impractical, even with the design of the sham acupuncture group. (b) The studies revealed that all current sham acupuncture modalities have positive therapeutic effects because they are not absolutely inert (59–61). Then, the therapeutic effect of acupuncture is easily underestimated when compared to sham acupuncture. Therefore, we have not designed a sham acupuncture group as the control. Nevertheless, the therapeutic effect may be influenced by the psychological effects coming from acupuncture, which should be interpreted in the study results.

## Conclusion

This protocol describes a single-center, assessor- and analyst-blinded prospective randomized controlled trial, which evaluates the safety and effectiveness of TNRM-UN for CV with deficiency of qi and blood. It ascertains whether TNRM-UN is more effective in current acupuncture therapies and can be used as a promising therapy for clinical practice.
